# Comparison Between Closed Suction Drainage and No Drainage Following Total Knee Arthroplasty in a Tertiary Care Setting in Pakistan

**DOI:** 10.7759/cureus.842

**Published:** 2016-10-24

**Authors:** Avais Raja, Hana Manzoor, Waqar M Jan, Salman Assad

**Affiliations:** 1 Department of Orthopaedic Surgery, Shifa College of Medicine, Islamabad, Pakistan; 2 Department of Neurology, Shifa International Hospital, Islamabad, Pakistan; 3 Department of Orthopaedic Surgery, Shifa International Hospital, Islamabad, Pakistan; 4 Department of Medicine, Shifa College of Medicine, Islamabad, Pakistan

**Keywords:** total knee arthroplasty, closed suction, no suction, allogeneic blood transfusion, pakistan

## Abstract

Background: Total knee arthroplasty is associated with hematoma formation and extensive blood loss up to 1.5 liters. The placement of a closed suction drain to control this complication is controversial. The purpose of this study is to determine the efficacy between total knee arthroplasty without a drain and with a closed suction drain.

Methods: A retrospective cohort study was conducted between January 2014 and December 2015 on 100 patients to assess the hemoglobin and hematocrit levels, the rate of allogeneic blood transfusion, and the length of hospital stay in patients with a closed suction drain versus no suction post-total knee arthroplasty.

Results: Fifty-six units of packed red blood cells were transfused in 36 out of 50 patients (72%) in the closed suction group compared to 21 units of packed red blood cells in 18 out of 50 patients (36%) in the no suction group after 24 hours post-surgery. The hemoglobin levels at multiple intervals were lower in the closed suction group (p < 0.05). The closed suction group also had an extra one-day stay in the hospital (p = 0.0492, 95% CI = 0.70 - 0.94).

Conclusion: Closed suction drain placement is associated with low hemoglobin levels, an increased rate of allogeneic blood transfusion, and a longer hospital stay.

## Introduction

Total knee arthroplasty, a common orthopedic reconstructive procedure, has helped many to overcome the disability and pain from osteoarthritis of the knee [1]. Despite the gratifying results, a major complication of hemorrhage is inevitable to prevent but can be controlled [2]. Many attempts to control the hemorrhage have been pursued, including femoral intramedullary plugs, use of fibrin, administration of antifibrinolytics, epinephrine, and closed suction drain [2]. However, a debate still remains on the application of a closed suction drain [3]. In its attempt to prevent hematoma formation, closed suction drainage promotes an additional risk of blood transfusion and lengthier hospital stay [4]. We have hereby performed a retrospective cohort study assessing the difference between the use of a closed suction drain and no drain on the rate of allogeneic blood transfusion, hemoglobin levels, and length of hospital stay. 

## Materials and methods

A total of 100 consecutive primary total knee arthroplasty procedures were performed between January 2014 and December 2015 by the senior consultants of our institution. Revision knee arthroplasties and unicompartmental knee arthroplasties were excluded. The standardized technique of total knee arthroplasty was performed by the senior surgeons. All procedures used a high thigh tourniquet, a midline incision, and a medial parapatellar approach. The femoral and tibial components were cemented, and the patellas were resurfaced. One type of prosthesis was used during this study: NexGen® (Zimmer, Ltd., Swindon, UK). The procedures were either performed with general anesthesia with an epidural or spinal anesthesia with an epidural. The tourniquet was deflated after the closure of the wound, application of dressings, and a compression bandage with the knee in extension. Routine deep vein thrombosis prophylaxis was comprised of thromboembolic deterrent stockings and Clexane, 10 mg injections once daily for six weeks unless contraindicated.

### Data collection

Data was collected retrospectively from records of procedures performed by two senior surgeons, where one senior surgeon utilized a closed suction drain and the other did not use a drainage system. Case notes were studied as well data on age, sex, BMI, type of anesthesia, tourniquet time, comorbidities, preoperative hematocrit and hemoglobin levels, postoperative hematocrit and hemoglobin levels (24-hour, 72-hour, and at discharge), the number of units of allogeneic packed red cells transfusions utilized, and the length of hospital stay. The surgeons obtained written informed consent from all the patients. There is no data that would reveal patient identity in any form in this report. 

### Statistical analysis

SPSS® 22.0 (SPSS, Chicago, IL, US) was used to analyze the data. Independent sample t-test for numerical variables and Pearson Chi-square (*X^2^) *were used to compute p-values. The threshold for statistical significance was p < 0.05 and was considered to be significant.

## Results

The closed suction drain group and no drain group were comprised of 50 patients each. No cases of infections or postoperative fractures were documented. There were 39 men and 61 female patients with a mean age of 62.11 ± 8.68 years ranging from 35 to 85 years of age.

### Hemoglobin levels and transfusion rates

In both closed suction drain and no drain groups, the mean preoperative hemoglobin was 12.76 ± 1.51 g/dL, the mean postoperative hemoglobin at 72 hours was 9.52 ± 1.95 g/dL, and the mean hemoglobin at discharge was 10.16 ± 1.07 g/dL. However, no statistical difference was found between the mean preoperative hemoglobin 12.48 ± 1.64 g/dL and 13.04 ± 1.33 g/dL for the closed suction drain group and the no drain group, respectively (p = 0.062) .

Overall, 77 units of packed red blood cells (PRBCs) were transfused into 53 patients in the 24 hour period following surgery. In the no drain group, 18/50 (36%) patients were transfused with a total of 21 units, while in the closed suction drain group, 36/50 (72%) patients were transfused with a total of 56 units. Hence, more patients were transfused, as well as more PRBCs transfusion units were required, in the closed suction drain group as compared to the no drain group (Pearson *X*^2^ = 18.21, p = 0.000398; Phi = 0.427, p = 0.000398). 

An independent t-test was conducted to compare the postoperative hemoglobin levels at 72 hours and at discharge as well as the length of hospital stay between the closed suction drain and the no drain groups. At 72 hours postoperatively, the mean hemoglobin value of 8.95 ± 1.17 g/dL for the closed suction drain group was less than the mean hemoglobin value of 10.09 ± 2.37 g/dL for the no drain group (p = 0.03, 95% CI = 0.39 - 1.88). Similarly, at discharge, the mean hemoglobin value of 9.94 ± 0.73 g/dL for the closed suction drain was less than the mean hemoglobin value of 10.38 ± 1.29 g/dL for the no drain group (p = 0.041, 95% CI = 0.17 - 0.86).

### Length of hospital stay

The mean duration of hospital stay for closed suction drain group was 6.84 ± 1.06 days, which was more than the mean duration for the no drain group at 5.72 ± 1.07 days of hospital stay (p = 0.0492, 95% CI = 0.70 - 0.94).

## Discussion

The use of a closed suction drain after orthopedic procedures to prevent hematoma production and wound problems was recommended by Waugh and Stinchfield in 1961 [5]. Nevertheless, it was challenged by many surgeons until a prospective study conducted by Beer, et al. in 1991 concluded postoperative wound drainage had no positive impact whatsoever on total knee arthroplasty [6].

The sole purpose of the closed suction drain is to reduce the amount of hematoma formation by discarding the collected blood [7]. However, this practice comes with a price, as observed in a meta-analysis conducted by Markar, et al. [8]. Their study revealed that closed suction drain after total knee arthroplasty was associated with an increase in homologous (allogeneic) blood transfusion rates and longer hospital stays [8]. Furthermore, homologous blood transfusions bring the risk of blood-borne infections (HIV, hepatitis B, hepatitis C), immunological problems (hemolytic reaction, allergic reaction, anaphylaxis), and cardiopulmonary complications (acute lung injury, transfusion-associated circulatory overload) [9]. The patients in our study were diagnosed cases of bilateral, end stage, advanced osteoarthritis. The surgeons, trained in a similar background, utilized the same technique and minimally constrained prosthesis in performing the total knee arthroplasty. The closed suction drain was inserted while closing the skin incision and removed after postoperative day 3. Hemoglobin and hematocrit levels were followed preoperatively and postoperatively at 24 hours, 72 hours, and discharge.

Hemoglobin and hematocrit levels were significantly low in the closed suction drain group compared to the no suction drain group, especially after 72 hours of operation. Ultimately, this group received a higher rate of homologous transfusions (72%) with 56 units of packed red blood cells in 36/50 patients after 24 hours of the procedure. Blood transfusion within 24 hours is not calculated in this figure as blood loss is immense from the cutting of the bone cortex and medulla intra-operatively. Although the donor blood was screened for common blood-borne infectious diseases meticulously in our institution, recipient patients were still subjected to screening to rule out HIV, hepatitis B, and hepatitis C. No blood-borne infections were documented in successive follow-ups. Only one report of an acute febrile hemolytic reaction was documented 20 minutes into the transfusion and the blood transfusion was halted.

Blood transfusion protocol varies from one institution to another. Although some research studies indicate transfusion below 8-10 g/dl, we individualized the blood transfusion criterion based on the age, clinical condition, and underlying comorbidities. Patients in the closed suction group had a longer stay in the hospital of one day in comparison to the no drain group. This can be explained by the additional need of blood transfusion and, consequently, close monitoring. Problems posed by blood loss in total knee arthroplasty and risks of allogeneic blood transfusion has researchers and surgeons desperate to find alternative methods to manage these issues. Recent methods under research are autologous transfusion, the use of an interval draining clamp, and the use of tranexamic acid [10-11]. These methods have reduced blood loss, hospital stay, and allogeneic blood transfusion rates post-total knee arthroplasty [10-11].

### Limitations

Our study is the first study of its kind conducted in Pakistan, to our knowledge. A retrospective study has its limitations; thus, a prospective trial should be conducted assessing the effectiveness of various methods to control blood loss post-total knee arthroplasty. The variability of findings and outcomes might have been affected due to Berksonian and selection biases. The external validation and generalization of this single center hospital experience cannot be applied to other population groups.

## Conclusions

Closed suction drain placement is associated with low hemoglobin levels, an increased rate of allogeneic blood transfusion, and a longer hospital stay. The reason behind the lower rate of allogeneic blood transfusion and higher hemoglobin levels in the group without drains may be attributed to the tamponade effect of the knee’s soft tissue encasing the joint cavity. 

## References

[1] Høvik LH, Winther SB, Foss OA, Gjeilo Gjeilo (2016). KH. Preoperative pain catastrophizing and postoperative pain after total knee arthroplasty: a prospective cohort study with one year follow-up. BMC.

[2] Madadi F, Mehrvarz AS, Madadi F, Boreiri M, Abachizadeh K, Ershadi A (2010). Comparison of drain clamp after bilateral total knee arthroplasty. J Knee Surg.

[3] Esler CN, Blakeway C, Fiddian NJ (2003). The use of a closed-suction drain in total knee arthroplasty. A prospective, randomised study. J Bone Joint Surg Br.

[4] Walmsley PJ, Kelly MB, Hill RM, Brenkel I (2005). A prospective, randomised, controlled trial of the use of drains in total hip arthroplasty. J Bone Joint Surg Br.

[5] Waugh TR, Stinchfield FE (1961). Suction drainage of orthopaedic wounds. J Bone Joint Surg Am.

[6] Beer KJ, Lombardi AV Jr, Mallory TH, Vaughn BK (1991). The efficacy of suction drains after routine total joint arthroplasty. J Bone Joint Surg Am.

[7] Cheung G, Carmont MR, Bing AJ, Kuiper JH, Alcock RJ, Graham NM (2010). No drain, autologous transfusion drain or suction drain? A randomised prospective study in total hip replacement surgery of 168 patients. Acta Orthop Belg.

[8] Markar SR, Jones GG, Karthikesalingam A, Segaren N, Patel RV (2012). Transfusion drains versus suction drains in total knee replacement: meta-analysis. Knee Surg Sports Traumatol Arthrosc.

[9] Levine BR, Haughom B, Strong B, Hellman M, Frank RM (2014). Blood management strategies for total knee arthroplasty. J Am Acad Orthop Surg.

[10] Singh VK, Singh PK, Javed S, Kumar K, Tomar J (2011). Autologous transfusion of drain contents in elective primary knee arthroplasty: its value and relevance. Blood Transfus.

[11] Chareancholvanich K, Siriwattanasakul P, Narkbunnam R, Pornrattanamaneewong C (2012). Temporary clamping of drain combined with tranexamic acid reduce blood loss after total knee arthroplasty: a prospective randomized controlled trial. BMC Musculoskelet Disord.

